# Exploring preventable lifestyle risk factors among newcomers in Montreal, Canada: a mixed-method study

**DOI:** 10.1093/heapro/daaf115

**Published:** 2025-07-15

**Authors:** Geneveave Barbo, Anissa Jeeroburkhan

**Affiliations:** College of Nursing, University of Saskatchewan, 107 Wiggins Rd., Saskatoon, Saskatchewan, Canada, S7N 2Z4; The Refugee Centre, 2107 Saint-Catherine St. West, Montreal, Quebec, Canada, H3H 1M6

**Keywords:** Transients and Migrants, newcomers, Healthy Lifestyle, Health Promotion, Quebec

## Abstract

Newcomers in Montreal, Quebec—including immigrants, international students, refugees, and asylum seekers—face lifestyle risk factors associated with chronic conditions. Gaps in the literature highlight methodological limitations in the previous studies as well as incomplete examination of physical activity, diet, smoking, alcohol use, and stress in this population. This study aims to examine these preventable lifestyle risk factors among Montreal newcomers and explore barriers and facilitators influencing them. Using a community-based participatory approach, we conducted a concurrent mixed-methods study, collecting data via surveys and focus groups. Survey data were analysed descriptively, and focus groups underwent thematic analysis. Among 149 survey and 55 focus group participants (equal gender distribution, mostly aged 18–29), engagement in physical activity varied. Barriers included weather, health issues, cultural adjustments, and lack of motivation; facilitators included social support and health concerns. Dietary habits favoured home-cooked meals, but significant fast-food consumption occurred due to time, cost, access, food quality challenges, dietary preferences, and nutritional awareness were facilitators. Smoking rates were low; many abstained from alcohol, with social influences as barriers and family support as facilitators. Stress levels were moderately high due to various pressures: coping strategies included therapy, physical activity, social support, and a positive mindset. Newcomers in Montreal display both healthy and risky lifestyle behaviours, with concerns around diet and stress. Targeted health promotion strategies addressing specific barriers and leveraging facilitators are needed to improve their health and well-being.

Contribution to Health Promotion

- This study identifies key lifestyle risks (physical activity, diet, smoking, alcohol use, and stress) among newcomers to Montreal affecting their long-term health.
- This study highlights both helpful factors and barriers that influence key lifestyle risk factors among newcomers.
- Based on this, the study findings offer insights into recommendations aimed at addressing these risk factors.

## INTRODUCTION

According to the most recent census conducted in 2016, 34% (approximately 644 680 individuals) of Montreal’s population are considered foreign-born, a trend expected to increase with Canada's new immigration plan for 2023–25 (Ville de Montréal 2021, Statistics Canada 2022, Government of Canada 2023). In 2022, Quebec welcomed over 68 700 permanent immigrants and 86 700 non-permanent residents who primarily settled in Montreal and other urban areas (Statistics Canada 2022, Institut de la statistique du Québec 2023). They were mostly from African (e.g. Algeria, Morocco, and Somalia), Asian (e.g. China, Lebanon, Philippines, and Vietnam), European (e.g. France, Italy, and Romania), and North American (e.g. Haiti) countries (Ville de Montréal 2021). This influx has highlighted the need to address health challenges among newcomers, particularly chronic conditions, such as cardiovascular diseases (CVD), diabetes, and certain cancers, linked to lifestyle risk factors such as diet, physical activity, smoking, alcohol consumption, and stress levels (Liu et al. 2010, Weisman et al. 2018, Leung et al. 2019, Adjei et al. 2020). In this paper, the term ‘newcomers’ is used to encompass immigrants, international students, refugees, and asylum seekers. This inclusive term emphasizes shared experiences of arrival, adaptation, and belonging, while intentionally avoiding the legal and political connotations associated with labels, such as ‘migrant’, and which do not align with the study’s focus on disease prevention.

Research indicates that newcomers to Canada, particularly those from South Asia and Somalia, face higher risks of these chronic conditions due to dietary habits, reduced physical activity, and heightened stress level, exacerbated by adaptation challenges, language barriers, and discrimination (Kilbride 2014, Rousseau and Frounfelker 2019, Anderson et al. 2022, Sebastian et al. 2022). Indeed, analysis of data from the Canadian Community Health Surveys (CCHS) (2001–18), a national cross-sectional survey performed by Statistics Canada annually, revealed that newcomers have greater prevalence of CVD risk factors compared with the Canadian-born population (Chen et al. 2024). The same study also demonstrates a considerable increase in diabetes and heavy alcohol consumption among newcomers (Chen et al. 2024). Additionally, lifestyle risk factors such as physical inactivity, obesity, and elevated cholesterol levels were more prevalent among newcomers (Chen et al. 2024). Using CCHS data focused on 2015–18, Lin (2024) also examined mood disorders quantitatively. This study presented that despite high levels of reported mental health challenges, newcomers were less likely to be diagnosed with mood or anxiety disorders (Lin 2024).

While these studies and the CCHS data offer crucial quantitative insights into the health disparities affecting newcomers, they often fail to capture the underlying causes and contextual factors of these disparities. Furthermore, newcomers may be hesitant to participate in government-conducted surveys due to concerns about potential repercussions on their immigration status, leading to an under-representation or social-desirability bias where respondents provide socially acceptable answers rather than their true experiences (Chen et al. 2024). Comparatively, Porto de Oliveira and Gosselin (2024) recently performed a qualitative study exploring the barriers and facilitators to sports and physical activity among immigrant and racialized families from disadvantaged neighbourhoods, as well as 2SLGBTQ + communities in Montreal. While the study identified lack of diversity, scheduling conflicts, and high costs as major obstacles, it had limitations—including an inadequately detailed methodology and a narrow focus that excluded other related lifestyle risk factors such as dietary habits, stress levels, and smoking and alcohol consumption (Porto de Oliveira and Gosselin 2024). Despite the valuable contributions of existing studies, there is a clear gap in the literature regarding the comprehensive exploration of lifestyle risk factors among newcomers in Montreal. Therefore, there is a pressing need for a comprehensive, community-driven, mixed-methods study that combines qualitative and quantitative approaches to thoroughly investigate these interconnected lifestyle risk factors.

## RESEARCH OBJECTIVES

In order to address the aforementioned literature gaps, the following research objectives guided this study: (i) to examine preventable lifestyle risk factors related to chronic illnesses among newcomers living in Montreal, Quebec, specifically their physical activity, diet and eating habits, smoking and alcohol consumption, and stress levels; and (ii) to explore barriers and facilitators influencing these preventable risk factors within such population.

## METHODS

### Research design

This study employed a concurrent mixed-methods design, guided by a community-based participatory research (CBPR) framework, to address its research questions comprehensively and ensure data triangulation. As such, data collection and analysis of both quantitative and qualitative data were performed simultaneously within the same timeframe. For the quantitative data, we utilized a cross-sectional survey design, while the qualitative data was collected through an interpretive phenomenological approach. CBPR has been described as ‘a collaborative approach to research that involves members of communities, academic researchers, practitioners, organizational representatives, and others in all aspects of the research process’ (Ward et al. 2018, p. 2). Grounded on the principles of equitable partnership, the CBPR framework assists researchers in recognizing the value of each stakeholder’s expertise and viewpoint in enhancing the various elements of research (Ward et al. 2018). This approach not only enriches the research process but also ensures that the outcomes are more pertinent and have a greater impact on the target population. These key tenets are crucial when conducting research with marginalized populations, as in the case of newcomers.

Following CBPR, an advisory committee (AC) was formed during the protocol development and the research ethics application phases. The AC comprised healthcare professionals (a doctor, nurse, and dietician), a researcher, and community support providers (organizers, coordinators, administrators, and directors) from diverse ethnic backgrounds and gender identities. Importantly, several AC members were newcomers themselves, contributing their firsthand experiences and ensuring that their perspectives informed and shaped every stage of the study.

Participating organizations included larger entities such as ‘PRAIDA—Regional Program for the Settlement and Integration of Asylum Seekers’, and the ‘CLSC de Parc-Extension’. Additional collaborators included several local community-based organizations and newcomer support centres who provided vital support throughout the project. The AC provided valuable input and involvement in developing the research protocol (including the creation and revision of non-standardized questionnaires), participant recruitment, data collection, and analysis processes. Notably, some newcomer AC members were actively engaged in participant recruitment and data collection.

### Ethics and participant recruitment

Before initiating participant recruitment and data collection, this study received research ethics approval from the Health Canada-Public Health Agency of Canada Research Ethics Board on 22 September 2022. We targeted newcomer adults, aged 18 years and older in Montreal, Quebec, who were accessing services from The Refugee Centre and other community partners involved in this study. Eligibility was limited to individuals who self-identified as newcomers and had arrived in Montreal within the 8 years preceding their recruitment. We did not collect detailed migration status, or exact length of stay. This was a deliberate choice, made with community partners, to avoid discomfort or perceived risk. Participants were recruited using convenience, purposive, and snowball sampling methods.

Recruitment efforts included distributing posters at The Refugee Centre following monthly health navigation workshops and weekly posts through the Centre’s social media channels. Additionally, AC members were encouraged to share these recruitment materials within their respective organizations. These recruitment efforts were conducted over the course of a year, following The Refugee Centre’s established process for engaging and reaching out to its community members. For the quantitative portion of the study, a sample size of 350 participants was aimed to reach representativeness. This target was derived using the formula $n=\frac{Z^{2}\;\times \;p\;\times \;(1-p)}{E^{2}}$, where *n* is the required sample size, *Z* is the *z*-score corresponding to a 95% confidence level, *P* is set to 60% based on the prevalence data from Chen et al. (2024) , and *E* is the margin of error at 5%. This specific formula was applied as it aligns with the study’s objectives and design. For the qualitative portion, recommended sample sizes for phenomenological approaches range from 1 to 325 (Creswell and Poth 2016). Therefore, to achieve rich and comprehensive findings that allow for adequate triangulation and corroboration, a desired sample of 325 was targeted. However, a smaller sample size may also be considered sufficient if data saturation is achieved. Saturation was considered achieved when additional focus groups no longer yielded new information relevant to the research questions (Creswell and Poth 2016).

### Quantitative methodology: cross-sectional survey design

Participants completed an electronic survey either through their devices or on computers provided at The Refugee Centre. Each section of the questionnaire was carefully constructed to measure variables aligned with key risk factors, including inadequate physical activity, poor dietary habits, smoking, alcohol consumption, and high stress levels. Socio-demographic questions provide context for better understanding these factors within specific population subgroups, such as gender, age, language, and minority status, which can influence health behaviours. Health and medical history questions (e.g. height, weight, and medical conditions) established baseline characteristics. Physical activity levels are assessed to evaluate the frequency and intensity of exercise habits. For dietary assessment, we used an adapted version of the Diet History Questionnaire (DHQ) and Food Frequency Questionnaire (FFQ) (Ng et al. 2018); while stress levels were measured using the Perceived Stress Questionnaire (PSQ) (Levenstein et al. 1993). For smoking and alcohol consumption, frequency, amount, and type were assessed.

The survey questions were revised based on feedback from the AC members. As a result, apart from the unmodified PSQ, the DHQ and FFQ were substantially refined to better suit the study’s objectives and the target population. These changes affected the psychometric properties of the DHQ and FFQ, limiting their ability to be directly compared with other studies and reducing the generalizability of findings. The major modifications focused on adjusting the questionnaires’ emphasis, format, and level of specificity.

Originally, the DHQ and FFQ are designed to assess detailed dietary intake over a specific period, typically requiring participants to report the frequency and portion sizes of a comprehensive list of food items (Ng et al. 2018). In contrast, our survey questions were designed to capture broader dietary behaviours and challenges relevant to the newcomer population in Montreal, focusing on patterns and barriers rather than detailed intake. For instance, questions such as ‘How would you rate your overall habits of eating healthy foods?’ and ‘How many times do you eat home-cooked meals a week?’ reflect subjective self-assessments and lifestyle trends rather than specific consumption data. Additionally, culturally relevant elements were incorporated, such as identifying common challenges in obtaining groceries, barriers to healthy eating, and cooking method preferences, such as baking or grilling.

These modifications, while reducing standardization, increased the relevance and applicability of the questionnaires to the target population. The detailed contents of the survey are available in Supplementary Appendix A. Data were analysed using descriptive statistics in SPSS, with any extreme or unrealistic responses excluded to ensure data integrity. All survey variables were measured using individual, standalone questions, with the exception of the PSQ. The PSQ score was calculated by averaging responses across its 10 items, each rated on a five-point Likert scale. This composite score was used to reflect participants’ overall perceived stress levels. All other data points were derived from single-item questions and reported individually.

### Qualitative methodology: interpretive phenomenology approach

Qualitative data were collected through focus group sessions that followed the interview guide which included open-ended questions that explored motivations and challenges related to physical activity, healthy eating, smoking, drinking, and stress management (see Supplementary Appendix B for detail). This guide was developed and refined through a review of pertinent literature and valuable contributions from the AC members (Tiedje et al. 2014, Dave et al. 2015, Curtin et al. 2018). Focus group sessions were conducted in a large, private room at The Refugee Centre after hours, facilitated by one to three trained research staff members. Notably, these staff members were predominantly recent newcomers to Montreal, having arrived approximately two years prior, and were also multilingual, fluent in Farsi, Arabic, Spanish, French, and English, which enriched the discussions.

Each session, guided by recommendations from Gill et al. (2008), included 3–10 participants and lasted 60–90 min. Participants could end the session earlier if desired, and a break with complimentary refreshments was offered. Sessions were audio-recorded, transcribed verbatim, and followed by a debriefing. This process entailed inquiring with participants about their experiences during the research, querying if they would be interested in receiving information about the study’s findings upon completion, and providing them with health promotion resources related to physical activity, healthy eating, smoking cessation, alcohol consumption reduction, and stress management.

Transcripts were thoroughly reviewed, and theoretical thematic analysis by Braun and Clarke (2006) was used to identify major themes and sub-themes. This specific type of thematic analysis was chosen due to its structured approach that aligns closely with the research objectives, which aim to explore predefined variables influencing lifestyle risk factors among newcomers in Montreal (Braun and Clarke 2006). To conduct this, the first author independently followed Braun and Clarke’s (2006) phases of thematic analysis: (i) familiarizing yourself with your data; (ii) generating initial codes; (iii) searching for themes; (iv) reviewing themes; (v) defining and naming themes; and (vi) producing the report. Throughout this process, the second author provided oversight and reviewed the content to ensure rigour.

After analysing both quantitative and qualitative data separately, findings were then integrated. All findings from the analysis—quantitative, qualitative, and integrated—were presented to the AC members. After reviewing the data, they offered their insights and feedback. Based on the AC members’ suggestions, certain aspects of our analysis were revisited to provide greater clarity and depth. This led to a more nuanced presentation of the data, capturing subtleties that enhanced the overall quality of the findings.

## RESULTS

A total of 149 participants completed the survey, and 55 participated in the focus group sessions. Of these, 40 participants took part in both the survey and the focus groups, while 15 attended only the focus groups. Despite not achieving the desired sample size for both the quantitative (i.e. *n* = 350) and qualitative portions (*n* = 325) of the study, valuable insights were gathered. For the quantitative portion, while the sample size is smaller than anticipated, it remains adequate to identify meaningful trends and relationships, particularly given the exploratory nature of the study. As for the qualitative component, data saturation was achieved after 55 participants, indicating that no new themes or insights were emerging. Subsequent focus group discussions reinforced the existing themes without contributing additional novel information. This indicated that we had reached data saturation, making further data collection unnecessary for the purposes of our study (Creswell and Poth 2016). The combination of these datasets, along with the overlap of 40 participants in both components, provides a strong basis for integrating quantitative and qualitative findings to address the research objectives comprehensively.

Moreover, complete data were obtained for core demographic variables such as gender, age, marital status, spoken languages, highest completed education, employment status, public health care coverage, and medical history. However, missing data occurred in several key areas, notably in the number of people living in a household, height, weight, hours of work per week, and alcohol consumed in a week. Specifically, the ‘People living in a household’ variable had 10 missing responses and 9 unrealistic or impossible values, indicating variability that required further analysis. In contrast, ‘Height’ and ‘Weight’ variables showed significant missing data and extreme values. ‘Height’ had 3 missing and 16 unrealistic or impossible values. While ‘Weight’ had 6 missing and 34 unrealistic or impossible values. Responses deemed unrealistic or physically impossible were excluded from the dataset. For example, in terms of height, entries such as 1.6, 1.69, 1.72, 1.75, 1.78 (listed twice), 1.8, 1.85, 5.2, 5.3, 5.7, 13.208, and 511 cm were omitted. For weight, any values below 90 lbs were classified as extreme outliers and consequently removed. Other variables that included unrealistic or impossible values are ‘Restaurant per week’ and ‘Meals per day’. One was noted in the former (−3 times/week), and three in the latter (−3, 0, and 21 meals/day). Survey responses are summarized in Supplementary Appendix C. Average completion time was 20–30 min.

The socio-demographic profiles of the survey and focus group participants indicate that men comprised 49% of survey respondents and 50.9% of focus group participants, while women accounted for 46.3% and 45.5%, respectively. Additionally, 2.7% of survey participants selected ‘other’ category, and 3.6% of focus group participants identified as non-binary. The participants were predominantly young, with the majority falling within the 18–29 age range (72.5% in the survey, 67.3% in the focus group), and most survey respondents reported being single (75%). Refer to Tables 1 and 2 for details.

**Table 1.** Socio-demographic profiles of survey participants.

Variables	*n* (%)
Gender	
Men	73 (49.0%)
Women	69 (46.3%)
Other	4 (2.7%)
Prefer not to disclose	3 (2.0%)
Age group	
18–29 years old	108 (72.5%)
30–44 years old	30 (20.1%)
45–58 years old	11 (7.4%)
Marital status	
Single	111 (75.0%)
Married	27 (18.2%)
Divorced/Separated	6 (4.0%)
Prefer not to disclose	4 (2.7%)

**Table 2.** Socio-demographic profiles of focus group participants.

Variables	*n* (%)
Gender	
Men	28 (50.9%)
Women	25 (45.5%)
Non-binary	2 (3.6%)
Age group	
18–29 years old	37 (67.3%)
30–44 years old	16 (29.1%)
45–58 years old	2 (3.6%)

### Quantitative findings

The study found that many participants engaged in some physical activity, with 84.4% having participated in sports or physical activities, 29.3% exercising one to two times per week, and 26.5% doing so three to four times per week. Despite the general engagement in physical activities, dietary habits presented challenges. Although the majority of respondents rated their eating habits as average (42.9%) to above average (34.0%) and half of them (50.4%) consumed home-cooked meals three to seven times per week, a notable portion relied heavily on fast food, fried items, or packaged snacks high in fats, salts, or sugars, with 29.7% consuming such foods one to two times daily. Consumption rates for sugary drinks and desserts were also high, reported at 33.1% and 31.1%, respectively for one to two times daily consumption.

Regarding smoking and alcohol consumption, only a small fraction of the participants smoked (12.8%), with cigarettes being the most common form of tobacco used. Alcohol consumption patterns varied widely among participants, with 44.7% abstaining from alcohol and 25% consuming alcohol about once a week.

In terms of stress levels, the PSQ analysis revealed that two responses had to be discarded due to significant missing data, while eleven underwent pro-rating for minor missing items. The results indicate a moderately high level of perceived stress among participants, with an average PSQ-Index of 0.7, a standard deviation of 0.1, and a range from 0.4 to 1.0. This suggests that although individual stress levels vary, they do so within a relatively narrow range.

### Qualitative findings

Five major themes emerged from the analysis qualitative data, each with their own sub-themes. They include: (i) negotiating limited means, (ii) not built for us, (iii) everything we carry, (iv) the drive to be better, and (v) making it work. Following Braun and Clarke’s (2024), themes were developed as shared patterns of meaning, not merely as topic categories.

#### Theme 1: negotiating limited means

*Unable to pay the price of wellness*. Many participants reported that financial limitations inhibit them from accessing to gyms and equipment, a challenge particularly pronounced among students and low-income individuals. Some suggested that the availability of free or subsidized physical activities, if supported by government initiatives, would likely boost participation. Economic considerations are similarly influential in dietary habits, with the high cost of fresh produce severely impacting food choices. Participants noted dramatic price differences compared to their home countries, making it difficult to afford fresh fruits, vegetables, and meat: ‘Because of the rising grocery prices that are just insane, it's really hard to keep up. Less and less meals are primarily meat and vegetable, and they're switching to more like lots of grain, lots of rice, noodles, [and] potatoes’ and ‘I think I would have my fridge full of fruits and vegetables, like really fresh, different cool kinds if I could but like the closest grocery store to me is IGA and they sell $10 of strawberries and it's just completely unethical’.

*Time constraints*. Another major challenge identified by most of the participants is the constraint of time, often due to demanding schedules involving work, studies, or other commitments. For instance, one person describes the difficulty in finding time for exercise while juggling two jobs, a full-time study load, and involvement in student clubs. Moreover, many are unable to find the time to go do grocery, prepare the food, cook, eat, and clean up, particularly those who have numerous academic and professional responsibilities, compelling many to opt for convenience foods or eating out as simpler, quicker alternatives. Indeed, as one participant explained, ‘Here I live alone, and I study. I work, I clean, I cook, I do the groceries and like, how do you manage to do that? So, I think you need to sacrifice something…either you go to the gym, or you have good grades or …I eat or I have my aid that I need to keep my scholarship to not disappoint everyone because I'm far away from everyone, so it's a game that there's no winners. You just lose and you lose’.

*Missing the familiar*. Accessibility to preferred or familiar foods is another hurdle. Many find that the diversity of vegetables, fruits, and spices they were accustomed to is lacking, leading to increased consumption of non-vegetarian and junk food, this was elaborated by one participant: ‘Here I am more relied on non-vegetarian food as compared to vegetarian and I'm eating more junk food as compared to my home country because of less availability of like vegetarian Dhaba style food in Canada’.

#### Theme 2: not built for us

*Weather and geography*. Weather conditions, particularly in colder climates, also emerge as a barrier. The extreme cold in Montreal, as mentioned by one respondent, discourages outdoor activities, including visits to the gym. Another participant divulged that the cold weather has been severely impacting their mental health to the point that they experience ‘seasonal depression here. There is no avoiding that, every year in winter’. Nevertheless, during summer season, participants are encouraged to perform physical activities. The city layout and access to exercise facilities, such as having a gym in the building or living in a walkable city, are also reported as facilitators. This is appropriately described by someone who noted the ease of running errands on foot in Montreal.

As for dietary habits and newcomers’ transition to the Canadian food markets, issues around the taste and quality of local produce frequently mentioned. Participants described vegetables and fruits in Montreal as less flavourful compared to those in their home countries, which impacts their willingness to purchase these items. Vegetables and fruits in Montreal were described by participants as ‘different’, ‘tasteless’, ‘watery’, ‘bland’, ‘weird’, ‘horrible’, and ‘rotten’. The larger portion sizes in Canada also pose a problem, as buying in bulk can lead to waste if the food is not consumed quickly.

Besides the differences in the availability of produce, certain participants also expressed their struggle adapting to their new environment, particularly when it regards needing to learn French from the ground up, as one participant explained: ‘This sense of not fitting into the society and perhaps if I moved to, I was just thinking about that the other day. Perhaps if I moved to an Anglophone province, it would be easier, you know, because and like, I feel more comfortable in English, in French, it's very challenging and people here, they are very crazy about French, which I don't like. But yeah, I don't know like mainly it's that I don't fit in society. I'm doing what I wanted to do and somehow I feel unsuccessful. That's my stress’.

*When belonging means bending*. Family, friends, and significant others greatly impact many participants’ physical activity levels, dietary habits, smoking and alcohol consumption, and overall stress. For example, social support is frequently cited as a key factor that encourages individuals to engage in physical activities, even when they lack the motivation to do so on their own. Conversely, the absence of social support can deter participants from initiating or maintaining a regular exercise regimen. Additionally, according to certain participants, the eating behaviours of family and peers are major influencers on the participants’ dietary choices, shaping their decisions regarding what and how they eat.

Furthermore, many participants noted that their smoking habits began as social activities influenced by peer pressure, as described by one participant: ‘Especially many of my friends like I can say 70% of my friends do smoking regularly. So, whenever I am out with them it is very difficult for me, and I need to be with them because whenever we go out for chill or something. Then they do it and I need to do the passive smoking because I can't stay away from them because of that habit’. This sentiment was echoed by others who felt that smoking or drinking was a way to fit in or be accepted in social circles. The ingrained nature of these habits in social and cultural contexts made quitting difficult for some.

Family and peer were also influential when it comes to alcohol consumption, as one participant noted, ‘If I had friends that didn't drink, I don't think I would drink or like the consumption would just stop … I don't drink alone. It's 99%, like almost always with people. So, it's either having friends that wouldn't drink, or some health problem would be the big push to stop consumption’. Many participants also had and have relatives and friends who were serious smokers or drinkers, which led them to encounter the negative health consequences of these substances firsthand, encouraging them not to follow the same path.

Additionally, familial separation exacerbated stress levels, with participants worrying about the well-being of family members in their home countries and experiencing guilt for being away. Many participants mentioned that they are continuously thinking about their families in their home country; they worry whether all their basic needs are met (e.g. food and shelter). This worry even further worsens when participants hear about tragic situations in their country from the media or their relatives, particularly since many are unable to leave Canada and assist their families even in cases where they are in danger.

Comparatively, social connections and support emerge as crucial facilitators to reducing stress, with one person emphasizing the importance of family and friends: ‘I am privileged to have a really great family and a really great friend group. I find it motivating to just like talking to them, let go of the psychological aspect of the stress, and then just like saying motivational words makes me feel a bit more calm’. Family and friends were identified as sources of support, motivation, and distraction, assisting in ‘offloading’ stress experienced by some participants. Lastly, some participants turned to religious and spiritual beliefs for solace and others adjusted their mindset, focusing on acceptance, and gratitude as key coping mechanisms.

#### Theme 3: everything we carry

*Perceptual influences*. Intrinsic factors, such as individual perceptions, also participants’ lifestyle behaviours. For instance, fears of judgement in gym settings and the effects of social media emerge as notable obstacles to engaging in physical activity. Additionally, some participants reported that they smoked cigarettes and drank alcohol more when they perceived that these would help boost their confidence to socialize with others more easily. This was further explained by some participants: ‘Alcohol boosts your confidence with girls …if you take it in excess, then it becomes a problem, but it just it boosts your ego and your confidence’ and ‘I don't necessarily say like in your daily life, I mean within that specific period of time with that specific group, you have this confidence among people to like, talk openly and just relay whatever message you want to relay so it's more of something that's happening at that time, not something that you do on a daily’.

Conversely, personal perceptions can also facilitate positive lifestyle habits. Some participants expressed a strong preference for eating fruits and vegetables, emphasizing that they have always enjoyed these types of food since they were young, and this is similar to ‘habits you formed’. As one participant asserted, ‘I eat them as a snack. They're I seek them out. It's not like I have to force myself. So I think that's a huge role in people eating or not eating vegetables and fruits is just enjoying them’.

*Immigration-related stressors*. Many participants reported that their stress primarily stems from academic demands, describing a relentless cycle of assignments and exams that parallels full-time workloads. The transition to a new country compounded this stress, especially with the pressure to excel academically to maintain scholarships and meet family expectations, as two participants emphasized: ‘I cannot let my parents down’ and ‘I am like exploding from stress because dude if I do not get a full score on that, like full, like one bad grade and they're going to deport me back to my country, you think that's not stressful?’

Furthermore, the uncertainty surrounding immigration and legal status is also a profound source of stress for many participants. Participants reported feeling stuck, anxious, and even afraid to leave their homes as they await the verdict of their immigration status application. This is explained by one of the participants: ‘Because at the end of the day, the difference between a permanent resident and a citizen is huge for me. I think it's just the fact that you might do everything right, but you start fearing that there's something you know you get racially profiled. For myself, it's not often, but it happens. It might happen and you never know. I sometimes I don't want to even step outside the house. You might not be doing anything, but you just don't want to step outside the house, it's not something that I want to go through with because I have lots of things to lose’. The stress associated with acquiring a status is even further aggravated by the long waiting time to get a response from the government, as one participant explained their frustrations: ‘It's my immigration status and it's not in my hands. It's up for the government to do what they do and that's not something that I can control… it's very difficult to live with this ambiguity about your future’.

#### Theme 4: the drive to be better

*Imagining a stronger self*. The pursuit of physical and mental well-being is strong driver for many participants, with individuals seeking exercise to improve their physical health and mental state. Specifically, mental resilience and stress reduction are key benefits sought from physical activity, with many viewing it as a means to enhance mental well-being. Certain participants recognize the importance of fitness for maintaining independence in later life and reducing illnesses, as echoed in the sentiment, ‘If we are not physically fit, then in the older age, we do not live the way we want… they can't do the work they want, they always need someone to help them’. While some participants also divulged that their physical and mental health issues hinder the ability to engage in regular exercise, making it even more important to gradually enhance their physical activity. These respondents note limitations due to health concerns, while others highlight mental health challenges like depression or anxiety that reduce their motivation for physical activity. For instance, one participant reported that having not performed any physical exercises in the past 5–6 years led to an increased difficulty to start any physical activity.

*Health awareness and education*. Health and nutritional awareness are prominently featured in the discussions. Participants express a keen understanding of the health benefits of their food choices, as captured in statements like, ‘The things which encourages me to have a lot of vegetables are the nutritional value they provide, they are healthy, they're rich in vitamins, they have minerals’ and the aesthetic appeal of vegetables, ‘The colorfulness of the vegetables energizes my kitchen’. Avoiding sickness and suffering, feeling healthy, mitigating the exacerbation of existing illnesses (e.g. eczema and diabetes), and living a long healthy life were also reported as health benefits to having an optimal diet and eating habits. Participants therefore recommended to have more educational initiatives to help newcomers understand the importance of nutrition and practical tips on preparing healthy meals affordably. This is elaborated by one participant: ‘Education is very much important and because with education, people will understand the content of what they are eating. You know, they will know that they'll have to mix the food, nutrients they eat. Proteins goes with carbohydrates and iron. So, if we have all that package on a daily basis, that's going to be fantastic to your health system, but with fast food, you only find when there is. You can't find all that. You know’.

Some participants also offered recommendations to encourage physical activity through social media, which revolve highlighting the mental and physical health benefits of regular exercise. Additionally, public health campaigns disseminated through movies, television advertisements, and packaging of the cigarettes were also noted by participants that facilitated their awareness and knowledge about the long-term health effects of alcohol and smoking consumption.

#### Theme 5: making it work

In response to the challenges of living a healthy lifestyle, participants have developed several adaptive strategies to overcome barriers such as high food costs and stress. Those facing affordability issues with food highlighted that they simply target foods that are on sale and constantly looking for cheaper options, even if they require going to multiple grocery stores. Specific programmes provided in schools also facilitate participants from acquiring affordable and healthy food options. Participants are also adapting by enhancing their cooking skills and experimenting with new recipes to better fit their new environment and maintain a healthy diet. Recommendations from the study include educational initiatives to help newcomers understand the importance of nutrition and practical tips on preparing healthy meals affordably.

For participants experiencing high levels of stress, a variety of coping mechanisms have been adopted, including therapy, medication, physical activity, hobbies and distractions, practical approaches, social support, religious and spiritual beliefs, mindset, and gratitude to manage these stressors. Certain participants highlighted the positive experience and benefits of going to therapy in reducing their stress levels. Some expressed how therapy assisted them to be more self-aware and ‘learn how to feel your feelings’ as well as ‘understand’ and ‘deal’ with their feelings. However, one participant expressed that therapy is still ‘not necessarily an immediate solution’. In addition to therapy, two participants mentioned using medications to alleviate their stress, but these medications only had short-term effects. Participants stated that ‘in time they did not reduce the stress’. Engaging in physical activities and hobbies, such as exercising, playing volleyball, practising yoga and meditation, and walking, offered a respite from stressors. While other participants preferred applying more practical strategies in dealing with their stress, such as determining their priorities, setting realistic targets, organizing their tasks accordingly, and adequately managing their time. Lastly, certain participants are more drawn to intrinsically applying changes in their mindset to deal with stress. For instance, some participants choose to completely avoid overthinking about the stressors in their lives and acknowledge that they are not as important or urgent as they initially seemed.

## DISCUSSION

This study provides a characterization of sociodemographic lifestyle risk factors related to chronic conditions among newcomers in Montreal, including physical activity, diet, smoking and alcohol use, and stress management, as well as an in-depth perspective of the barriers and facilitators to improve these behaviours.

Participants in our study demonstrated higher levels of physical activity compared to previous findings by Chen et al. (2024), which revealed over 50% of racialized recent immigrants were physically inactive. This discrepancy suggests that the level of physical activity among immigrants can vary significantly based on various demographic factors. These may include the duration of time since immigration, socioeconomic status, cultural integration processes within different immigrant communities, and geographical location (Chen et al. 2024). Fortunately, the qualitative portion of this study allows for a more nuanced understanding of the context. This approach identified similarities with the findings of Porto de Oliveira and Gosselin (2024), who noted scheduling conflicts and high costs as barriers to engaging in sports and physical activity. Participants in this study further expanded on these barriers, adding adverse weather conditions, physical and mental health challenges, cultural adjustments, and lack of motivation as additional obstacles.

In regard to diet and eating habits, although participants expressed a strong preference for home-cooked meals, many still resorted to consuming fast food and unhealthy snacks, indicating a tension between their dietary intentions and actual eating habits. This inconsistency may be attributed to major challenges in maintaining healthy diets reported by the participants, such as time constraints, high costs, limited access to healthy foods, and concerns about food quality and quantity. These barriers ultimately point to the presence of food insecurity. Food insecurity has been characterized as ‘inadequate access to safe and nutritious food to meet dietary needs and food preferences for an active and healthy life’ (Davison and Gondara 2021). Chevrier et al. (2023) have also pointed out similar high levels of food insecurity among Syrian refugee households resettled in Quebec. Likewise, a scoping review conducted by Jefferies et al. (2022) asserts the moderate to severe levels of food insecurity in newcomer African Canadian communities, particularly those who reside in Ontario and Quebec.

Regarding smoking and alcohol consumption, a relatively low percentage of participants engage in these behaviours among this study’s participants. This contrasts with Chen et al. (2024), who observed an increase of prevalence of heavy drinking among nonracialized established immigrants and a decrease in current smoking across all groups, including both recent and established immigrants. These contrasting findings may be influenced by several factors. For example, Chen et al.’s (2024) focus on longitudinal changes offers insights into trends over time, which are not captured in our cross-sectional study design. Moreover, this comparison might suggest that the Canadian public health initiatives targeting smoking cessation could be having a uniform impact across newer and established populations, or it may reflect a broader societal shift away from smoking behaviours.

Moderately high stress levels reported by participants, driven by academic, professional, and migration-related pressures, highlight significant mental health challenges within this population. In addition to these barriers, a notable link exists between food insecurity and mental health conditions, including depression and anxiety (Pourmotabbed et al. 2020, Tribble et al. 2020, Davison and Gondara 2021). Having high levels of stress has been shown to be associated with decreased intake of healthy foods, and increased intake of unhealthy foods, according to the meta-analysis performed by Hill et al. (2022). Similar findings have been reported by participants in this study. Despite the recognized connection between food insecurity, mental health difficulties, and unhealthy lifestyle choices among newcomers, there is a lack of interventions targeting these issues concurrently. A culturally tailored programme to address healthy eating, food insecurity, and mental health among newcomers in Montreal is therefore vital.

### Strengths and limitations

This study offers comprehensive insights into the lifestyle risk factors among Montreal newcomers, while leveraging a community participatory approach. However, it is important to acknowledge certain methodological limitations. These include the use of non-standardized questionnaires created with community partner feedback and an inadequate sample size that may not be representative of the broader population. This approach limited the scope to descriptive analysis and affected the results’ generalizability. Due to challenges encountered in the recruitment process, our study was completed with 149 participants, significantly below the initially targeted sample size of 350. This shortfall has raised concerns regarding the statistical power of the study and the breadth of the confidence intervals, potentially limiting the robustness and the precision of the findings. Moreover, the reduced sample size may have affected the representativeness of the study population, introducing a bias that could skew the results and limit their applicability to the broader population. While efforts were made to ensure that the sample included a diverse participant base, the smaller number may not fully capture the variability, and nuances present within the larger community. This limitation is important to consider when interpreting the results, as it may influence the generalizability of the study's conclusions. Despite these challenges, the study demonstrated a strong commitment to community engagement through effective partnership with local community organizations, providing valuable insights into lifestyle risk factors among a scarcely studied group.

Another limitation is the absence of participants’ migration status data and the number of years they had lived in Montreal. These omissions limit the depth of analysis. We also acknowledge that the newcomer population is highly heterogeneous, and that factors such as language proficiency, education, and employment influence health experiences. However, small subgroup sizes prevented disaggregated analysis, as it could compromise confidentiality and lead to unreliable comparisons. While findings are presented in aggregate, this may mask important within-group differences. Future research with larger, stratified samples should address these complexities using ethical and robust methods.

For the qualitative portion, the study benefited from the use of focus groups. Although the nature of focus groups risked overshadowing less vocal participants, the facilitators—who were newcomers themselves and spoke the participants’ languages—ensured inclusive and culturally sensitive discussions, with interpreters on hand to overcome any language barriers.

## CONCLUSION

Our study found that newcomers in Montreal exhibit both healthy and risky lifestyle behaviours, with notable concerns in dietary habits and stress levels. Key barriers included logistical and financial constraints, cultural adjustments, and mental health issues; while facilitators involved community engagement and culturally appropriate resources. These findings lay the groundwork for a more inclusive, holistic health promotion approach for such population. Future research should aim for more representative sample to enhance the validity and explore longitudinal trends to guide programme development. It is also essential to refine and evaluate the effectiveness of these programmes, while focusing on their long-term impacts on newcomers.

Furthermore, a deeper exploration into the intersectionality of factors such as gender, age, socioeconomic status, and immigration status, is required to understand fully the complexity of health behaviours and outcomes among such populations. Policies that improve access to affordable, healthy food options and safe, accessible mental health support are also essential to promote the health and well-being of these communities. By addressing the unique needs and challenges faced by these populations, we can work towards a healthier, more equitable society for all.

## Supplementary Material

daaf115_Supplementary_Data

## Data Availability

The data underlying this article cannot be shared publicly due to the privacy of individuals who participated in the study. The data will be shared on reasonable request to the corresponding author.
