# Supplementary material for: Exploring preventable lifestyle risk factors among newcomers in Montreal, Canada: a mixed-method study
Source: Health Promot Int. 2025 Jul 15;40(4):daaf115. doi: 10.1093/heapro/daaf115 (PMC12261908; doi:10.1093/heapro/daaf115)
Supplement: daaf115_Supplementary_Data [file daaf115_supplementary_data.docx]

# Appendix A

Questionnaires

Socio-demographic Questions

What is your age?

[Short Answer] years old

What is your gender?

[Man]

[Woman]

[Other]

[I do not wish to disclose]

What are the first characters of your postal code (A postal code is made up of 6 digits and letters, for ex: X0X 7Y8, only share X0X)

[Short Answer]

What is your marital status?

[Married]

[Divorced/Separated]

[Single]

[Prefer not to answer]

What are the languages you speak?

[English]

[French]

[Spanish]

[Arabic]

[Farsi]

[Other: please specify]

What are the languages you understand?

[English]

[French]

[Spanish]

[Arabic]

[Farsi]

[Other: please specify]

What are the languages you can read?

[English]

[French]

[Spanish]

[Arabic]

[Farsi]

[Other: please specify]

Do you consider yourself a visible minority?

[Yes]

[No]

[Prefer not to answer]

Including you, how many people live in your household?

[Short Answer]

Are there children under 18 years of age that live in your household?

[Yes]

[No]

[Prefer not to answer]

How much schooling did you complete?

[Did not finish high school]

[Gradated high school]

[Some college/university]

[College diploma/certificate]

[Professional degree]

[Undergraduate degree]

[Masters/graduate degree]

[Doctorate]

What is your employment status?

[Employed full time]

[Employed part time]

[Self-employed]

[Homemaker]

[Student]

[Looking for a job]

[Leave of absence]

[Uninterested in working]

How many hours do you work a week?

[Short Answer]

Do you have health care coverage with your employer?

[Yes]

[No]

[Not applicable]

Are you currently covered under the health insurance of the government?

[Yes]

[No]

If you do know, what does your insurance cover or not cover?

[Short Answer]

Health and medical history

What is your height?

[Short Answer] cm

What is your weight?

[Short Answer] lbs

Have you ever been told by a doctor or health care practitioner that you have heart disease or are at risk of?

[Yes]

[No]

Have you ever been told by a doctor or health care practitioner that you have diabetes or are at risk of?

[Yes]

[No]

Do you have a family history of heart disease?

[Yes]

[No]

Physical Activity

Please answer the following questions related to what an average week looks like for you. If you are consistent with your habits, use the last week as your point of reference.

How many days a week do you exercise or do some kind of physical activity for 30 minutes or longer? (This can include physical activity at your job

[None - 0 times per week]

[Occasionally]

[1-2 times per week]

[3-4 times per week]

[5 and more]

Have you ever played a sport or participated in physical activity?

[Yes]

[No]

Diet and Eating Habits

Please answer the following questions related to what an average week looks like for you. If you are consistent with your habits, use the last week as your point of reference.

How would you rate your overall habits of eating healthy foods? Answer based on a scale of 1-5, where 1 is poor and 5 is excellent:

[1] Poor

[2] Below Average

[3] Average

[4] Above Average

[5] Excellent

How many times do you eat home-cooked meals a week?

[Short Answer]

How many times do you eat at a restaurant a week?

[Short Answer]

How many meals do you eat in a day?

[Short Answer]

Why do you think that is?

[Short Answer]

What is your biggest meal of the day?

[Short Answer]

How many times a day do you eat fast/fried food/or packaged snacks, like potato chips, high in fat/salt/or sugar?

[None]

[Occasionally]

[1-2 times a day]

[3-4 times a day]

[5 and more]

How many regular soda, sweet tea, juice, energy/sports drinks, sweetened-coffee or other sugar-sweetened beverages do you drink each day?

[None]

[Occasionally]

[1-2 times a day]

[3-4 times a day]

[5 and more]

How many times a day do you eat sweet foods or desserts, like chocolate or ice cream, and other sweets?

[None]

[Occasionally]

[1-2 times a day]

[3-4 times a day]

[5 and more]

How many times a day do you eat dairy products (milk, unsweetened yogurt, low fat cheese)?

[None]

[Occasionally]

[1-2 times a day]

[3-4 times a day]

[5 and more]

How many times a day do you eat meat/poultry/fish?

[None]

[Occasionally]

[1-2 times a day]

[3-4 times a day]

[5 and more]

How many servings of fresh, canned, frozen, or dried vegetables do you eat each day?

[None]

[Occasionally]

[1-2 times a day]

[3-4 times a day]

[5 and more]

How often do you bake or grill your food instead of frying?

[Never or almost never]

[Sometimes]

[Most of the time]

[All of the time]

Do you find it challenging to get the groceries you need to prepare your meals?

[Yes]

[No]

[Sometimes]

What is the biggest challenge? Select all that apply.

[Distance to the grocery store/lack of transportation]

[Difficulty in finding the right ingredients]

[Cost of ingredients is too expensive]

[Other: please specify]

Smoking and Alcohol Consumption

Please answer the following questions related to what an average week looks like for you. If you are consistent with your habits, use the last week as your point of reference.

Do you currently smoke or ingest any other form of tobacco?

[Yes]

[No]

In what form do you ingest/consume tobacco? (e.g., smoking or chewing)

[Short Answer]

If you do smoke, how many cigarettes/packs do you smoke in a week? (please specify if the answer is for the amount of cigarettes or packs)

[Short Answer]

How often do you ask people to NOT smoke in your home?

[Never or almost never]

[Sometimes]

[Most of the time]

[All of the time]

[No one ever smokes in my home]

How often do you consume alcohol (1 glass of wine, 1 ounce of hard liquor, or 1 beer)l in a week? (please specify if the answer is for glass of wine, once of hard liquor, or beer)

[Short Answer]

Stress Levels

For each sentence, please select a number that best describes how often this applies to you

You feel rested

[1. Never]

[2. Sometimes]

[3. Often]

[4. Always]

You feel that too many demands are being made on you

[1. Never]

[2. Sometimes]

[3. Often]

[4. Always]

You are annoyed easily

[1. Never]

[2. Sometimes]

[3. Often]

[4. Always]

You have too many things to do

[1. Never]

[2. Sometimes]

[3. Often]

[4. Always]

You feel lonely or isolated

[1. Never]

[2. Sometimes]

[3. Often]

[4. Always]

You find yourself in situations of conflict

[1. Never]

[2. Sometimes]

[3. Often]

[4. Always]

You feel you're doing things you really like

[1. Never]

[2. Sometimes]

[3. Often]

[4. Always]

You feel tired

[1. Never]

[2. Sometimes]

[3. Often]

[4. Always]

You fear you may not manage to attain your goals

[1. Never]

[2. Sometimes]

[3. Often]

[4. Always]

You feel calm

[1. Never]

[2. Sometimes]

[3. Often]

[4. Always]

You have too many decisions to make

[1. Never]

[2. Sometimes]

[3. Often]

[4. Always]

You feel frustrated

[1. Never]

[2. Sometimes]

[3. Often]

[4. Always]

You are full of energy

[1. Never]

[2. Sometimes]

[3. Often]

[4. Always]

You feel tense

[1. Never]

[2. Sometimes]

[3. Often]

[4. Always]

Your problems seem to be increasing

[1. Never]

[2. Sometimes]

[3. Often]

[4. Always]

You feel you're in a hurry

[1. Never]

[2. Sometimes]

[3. Often]

[4. Always]

You feel safe and protected

[1. Never]

[2. Sometimes]

[3. Often]

[4. Always]

You have many worries

[1. Never]

[2. Sometimes]

[3. Often]

[4. Always]

You are under pressure from other people

[1. Never]

[2. Sometimes]

[3. Often]

[4. Always]

You feel discouraged

[1. Never]

[2. Sometimes]

[3. Often]

[4. Always]

You enjoy yourself

[1. Never]

[2. Sometimes]

[3. Often]

[4. Always]

You are afraid for the future

[1. Never]

[2. Sometimes]

[3. Often]

[4. Always]

You feel you're doing things because you have to, not because you want to

[1. Never]

[2. Sometimes]

[3. Often]

[4. Always]

You feel critized or judged

[1. Never]

[2. Sometimes]

[3. Often]

[4. Always]

You are lighthearted

[1. Never]

[2. Sometimes]

[3. Often]

[4. Always]

You feel mentally tired

[1. Never]

[2. Sometimes]

[3. Often]

[4. Always]

You have trouble relaxing

[1. Never]

[2. Sometimes]

[3. Often]

[4. Always]

You feel that you have to much responsibility

[1. Never]

[2. Sometimes]

[3. Often]

[4. Always]

You have enough time for yourself

[1. Never]

[2. Sometimes]

[3. Often]

[4. Always]

You feel under pressure from deadlines

[1. Never]

[2. Sometimes]

[3. Often]

[4. Always]

Appendix B

Interview Guide

**Physical activity**

- Describe your experience with physical activity during the last year
  - Do you exercise regularly?
- What do you think impacts your ability to do physical activity?
  - What encourages or discourages you to exercise?
  - What are the biggest barriers or challenges to your practice of physical activity?
  - What has helped or motivated you to perform physical activity?
- Do you feel like you have the physical capacity to exercise?
- Do you feel like you have time to exercise?

**Diet and eating habits**

- Please describe your diet and eating habits during the last year
  - Do you think you eat healthy? Are you satisfied with the way you eat?
- What are the biggest barriers or challenges to your consumption of fruits and vegetables?
  - What are the barriers or challenges that stop you from eating healthy?
  - Is there something that prevents you from eating healthy?
  - Do you find you have access to the groceries you need to cook at home?
- What has helped or motivated you to enhance your consumption of fruits and vegetables?
  - What can help or motivate you to eat healthily?

**BREAK TIME**

**Smoking and alcohol consumption**

- Describe your experience with smoking and/or alcohol consumption during the last year
  - Do you or have you ever smoked before? What led you to start smoking?
- Do people around you smoke? Does it bother you?
- What are the biggest barriers or challenges to the reduction of your smoking and/or alcohol consumption?
- What has helped or motivated you to reduce your smoking and/or alcohol consumption?

**Stress level**

- Please describe your stress level during the last year
- What are the biggest barriers or challenges to the reduction of your stress level?
- What has helped or motivated you to reduce your stress level?

**Recommendations to address barriers**

- How can the barriers you have previously described can be addressed? Any recommendations or suggestions?
- In your opinion, what kind of interventions would be best for encouraging physical activity? Healthy eating? The reduction of smoking and/or alcohol consumption?

**Other**

- Would you like to share anything else about your experience in improving your health /and lifestyle behaviours that we have not discussed today?

Appendix C

Survey Response Summary

| Variables | *n* (%) / *M* (*SD)* [Range] |
| --- | --- |
| Gender |  |
| Men | 73 (49.0%) |
| Women | 69 (46.3%) |
| Other | 4 (2.7%) |
| Prefer not to disclose | 3 (2.0%) |
| Age group |  |
| 18-29 years old | 108 (72.5%) |
| 30-44 years old | 30 (20.1%) |
| 45-58 years old | 11 (7.4%) |
| Marital status |  |
| Single | 111 (75.0%) |
| Married | 27 (18.2%) |
| Divorced/Separated | 6 (4.0%) |
| Prefer not to disclose | 4 (2.7%) |
| Self-identified as visible minority |  |
| Yes | 65 (45.1%) |
| No | 67 (46.5%) |
| Prefer not to answer | 12 (8.3%) |
| Spoken languages |  |
| English only | 22 (14.8%) |
| English and one additional language |  |
| English and Arabic | 5 (3.4%) |
| English and Farsi | 9 (6.0%) |
| English and French | 10 (6.7%) |
| English and Spanish | 15 (10.1%) |
| English and Other | 43 (28.9%) |
| English and two additional languages |  |
| English, Arabic, and French | 5 (3.4%) |
| English, Farsi, and Other | 2 (1.3%) |
| English, French, and Farsi | 4 (2.7%) |
| English, Spanish, and French | 12 (8.1%) |
| English, French, and Other | 9 (6.0%) |
| English, Spanish, and Other | 3 (2.0%) |
| English and three additional languages |  |
| English, Arabic, French, and Other | 1 (0.7%) |
| English, Spanish, French, and Arabic | 2 (1.3%) |
| English, French, Spanish, and Other | 1 (0.7%) |
| French only | 1 (0.7%) |
| Spanish only | 4 (2.7%) |
| Other only | 1 (0.7%) |
| Understood languages |  |
| English only | 22 (14.8%) |
| English and one additional language |  |
| English and Arabic | 2 (1.4%) |
| English and Farsi | 11 (7.5%) |
| English and French | 13 (8.8%) |
| English and Spanish | 10 (6.8%) |
| English and Other | 34 (23.1%) |
| English and two additional languages |  |
| English, Arabic, and French | 7 (4.8%) |
| English, Farsi, and Other | 2 (1.3%) |
| English, French, and Farsi | 3 (2.0%) |
| English, Spanish, and French | 17 (11.6%) |
| English, French, and Other | 8 (5.4%) |
| English and three additional languages |  |
| English, Arabic, French, and Other | 1 (0.7%) |
| English, Spanish, French, and Arabic | 2 (1.4%) |
| English, French, Spanish, and Other | 6 (4.1%) |
| English, French, Farsi, and Other | 1 (0.7%) |
| English and four additional languages |  |
| English, Spanish, French, Farsi and Other | 1 (0.7%) |
| French only | 1 (0.7%) |
| Spanish only | 5 (3.4%) |
| Farsi only | 1 (0.7%) |
| Other only | 1 (0.7%) |
| Read languages |  |
| English only | 29 (19.7%) |
| English and one additional language |  |
| *English and Arabic* | *2 (1.4%)* |
| *English and Farsi* | *9 (6.1%)* |
| *English and French* | *14 (9.5%)* |
| *English and Spanish* | *10 (6.8%)* |
| *English and Other* | *29 (19.7%)* |
| English and two additional languages |  |
| *English, Arabic, and French* | *8 (5.4%)* |
| *English, Farsi, and Other* | *1 (0.7%)* |
| *English, French, and Farsi* | *5 (3.4%)* |
| *English, Spanish, and French* | *21 (14.3%)* |
| *English, French, and Other* | *4 (2.7%)* |
| English and three additional languages |  |
| *English, Arabic, French, and Other* | *1 (0.7%)* |
| *English, Spanish, French, and Arabic* | *2 (1.4%)* |
| *English, Spanish, French, and Farsi* | *1 (0.7%)* |
| *English, French, Spanish, and Other* | *5 (3.4%)* |
| French only | 2 (1.4%) |
| Spanish only | 4 (2.7%) |
| Postal codes |  |
| Brossard | 1 (0.7%) |
| Côte Saint-Luc | 8 (5.6%) |
| Côte-des-Neiges–Notre-Dame-de-Grâce | 21 (14.7%) |
| Dollard-des-Ormeaux | 1 (0.7%) |
| Dorval | 1 (0.7%) |
| La Prairie | 1 (0.7%) |
| LaSalle | 1 (0.7%) |
| Laval | 2 (1.4%) |
| Mercier–Hochelaga-Maisonneuve | 4 (2.8%) |
| Montreal West | 1 (0.7%) |
| Montréal-Nord | 1 (0.7%) |
| Mount Royal | 3 (2.1%) |
| Outremont | 2 (1.4%) |
| Plateau-Mont-Royal | 12 (8.4%) |
| Rosemont–La Petite-Patrie | 2 (1.4%) |
| Saint-Laurent | 3 (2.1%) |
| Sud-Ouest | 2 (1.4%) |
| Verdun | 7 (4.9%) |
| Ville-Marie | 65 (45.5%) |
| Villeray–Saint-Michel–Parc-Extension | 3 (2.1%) |
| Westmount | 1 (0.7%) |
| People living in a household^a^ |  |
| 1-2 residents | 63 (48.5%) |
| 3-4 residents | 56 (43.1%) |
| 5-6 residents | 11 (8.5%) |
| People living with children^b^ | 17 (11.4%) |
| Prefer not to answer | 2 (1.3%) |
| Highest completed education |  |
| Did not finish high school | 2 (1.3%) |
| Graduated high school | 22 (14.8%) |
| Some college / university | 26 (17.4%) |
| College diploma / certificate | 8 (5.4%) |
| Professional degree | 5 (3.4%) |
| Undergraduate degree | 38 (25.5%) |
| Masters / Graduate degree | 47 (31.5%) |
| Doctorate | 1 (0.7%) |
| Employment status |  |
| Employed full-time | 13 (8.7%) |
| Employed part-time | 28 (18.8%) |
| Self-employed | 2 (1.3%) |
| Student | 79 (53.0%) |
| Homemaker | 1 (0.7%) |
| Looking for work | 25 (16.8%) |
| Uninterested in working | 1 (0.7%) |
| Hours of work per week |  |
| More than 40 hours (over full-time) | 3 (2.1%) |
| 35-40 hours (full-time) | 13 (9.2%) |
| up to 34 hours (part-time) | 64 (45.1%) |
| 0 hours (no work or not applicable) | 62 (43.7%) |
| Employment health care coverage |  |
| Have coverage | 29 (19.6%) |
| Do not have coverage | 64 (43.2%) |
| Not applicable | 55 (37.2%) |
| Public health care coverage |  |
| Have coverage | 58 (38.9%) |
| Do not have coverage | 91 (61.1%) |
| Height (cm) | 169.1 (9.9) [150-200] |
| Weight (lbs) | 148.0 (34.4) [90-240) |
| Medical history |  |
| Diagnosis or risk of DM | 12 (8.1%) |
| Diagnosis or risk of heart disease | 4 (2.7%) |
| Physical activity per week^c^ |  |
| None | 13 (8.8%) |
| Occasionally | 37 (25.2%) |
| 1-2 times | 43 (29.3%) |
| 3-4 times | 39 (26.5%) |
| 5 and more times | 15 (10.2%) |
| Participated in sports or physical activity |  |
| Have participated | 124 (84.4%) |
| Have not participated | 23 (15.6%) |
| Rating for overall healthy eating habits |  |
| Excellent | 9 (6.1%) |
| Above average | 50 (34.0%) |
| Average | 63 (42.9%) |
| Below average | 21 (14.1%) |
| Poor | 4 (2.7%) |
| Home cooked meals consumption per week |  |
| 0-2 times | 10 (7.1%) |
| 3-7 times | 71 (50.4%) |
| 8-14 times | 37 (26.2%) |
| 15-21 times | 23 (16.3%) |
| Restaurant per week |  |
| 0-1 times | 87 (62.1%) |
| 2-3 times | 35 (25.0%) |
| 4-5 times | 11 (7.9%) |
| 6 and more times | 7 (5.0%) |
| Meals per day |  |
| 1 meal | 5 (3.9%) |
| 2-2.5 meals | 56 (43.4%) |
| 3-3.5 meals | 57 (44.2%) |
| 4 meals and more times | 11 (8.5%) |
| Biggest meal of the day |  |
| Breakfast | 9 (6.2%) |
| Lunch | 59 (40.4%) |
| Dinner | 67 (45.9%) |
| Vary | 5 (3.4%) |
| Other | 6 (4.1%) |
| Daily consumption of fast/fried food/or packaged snacks high in fat/salt/or sugar |  |
| 1-2 times | 44 (29.7%) |
| 3-4 times | 6 (4.1%) |
| 5 and more | 1 (0.7%) |
| Occasionally | 84 (56.8%) |
| None | 13 (8.8%) |
| Daily consumption of regular soda, sweet tea, juice, energy/sports drinks, sweetened-coffee or other sugar sweetened beverages |  |
| 1-2 times | 49 (33.1%) |
| 3-4 times | 6 (4.1%) |
| 5 and more | 3 (2.0%) |
| Occasionally | 54 (36.5%) |
| None | 36 (24.3%) |
| Daily consumption of sweet foods or desserts, like chocolate or ice cream, and other sweets |  |
| 1-2 times | 46 (31.1%) |
| 3-4 times | 3 (2.0%) |
| 5 and more | 3 (2.0%) |
| Occasionally | 84 (56.8%) |
| None | 12 (8.1%) |
| Daily consumption of dairy products (milk, unsweetened yogurt, low fat cheese) |  |
| 1-2 times | 79 (53.4%) |
| 3-4 times | 10 (6.8%) |
| 5 and more | 6 (4.1%) |
| Occasionally | 46 (31.1%) |
| None | 7 (4.7%) |
| Daily consumption of meat/fish/beans |  |
| 1-2 times | 67 (45.6%) |
| 3-4 times | 15 (10.2%) |
| 5 and more | 4 (2.7%) |
| Occasionally | 43 (29.3%) |
| None | 18 (12.2%) |
| Daily consumption of fresh, canned, frozen or dried vegetables |  |
| 1-2 times | 76 (51.7%) |
| 3-4 times | 10 (6.8%) |
| 5 and more | 3 (2.0%) |
| Occasionally | 49 (33.3%) |
| None | 9 (6.1%) |
| Frequency of baking or grilling instead of frying |  |
| Never or almost never | 20 (13.5%) |
| Sometimes | 65 (43.9%) |
| Most of the time | 42 (28.4%) |
| All of the time | 21 (14.2%) |
| Experienced challenge obtaining grocery |  |
| Yes | 48 (32.4%) |
| Sometimes | 54 (36.5%) |
| No | 46 (31.1%) |
| Request others to not smoke |  |
| All of the time | 12 (8.3%) |
| Most of the time | 10 (6.9%) |
| Sometimes | 13 (9.0%) |
| Never or almost never | 37 (25.5%) |
| No one ever smokes in my home | 73 (50.3%) |
| Tobacco consumption | 19 (12.8%) |
| Form of tobacco consumed |  |
| Cigarette | 14 (23.3%) |
| Shisha | 2 (3.3%) |
| Vape | 1 (1.7%) |
| Not applicable | 43 (71.7%) |
| Cigarettes smoked in a week |  |
| 1-5 cigarettes | 8 (13.3%) |
| 6-10 cigarettes | 1 (1.7%) |
| 11 and more cigarettes | 5 (8.3%) |
| Not applicable or none | 46 (76.7%) |
| Alcohol consumption in a week |  |
| 4 or more times | 6 (4.5%) |
| 2-3 times | 11 (8.3%) |
| Once | 33 (25.0%) |
| Bi-weekly or monthly | 10 (7.6%) |
| Occasional or inconsistent | 13 (9.8%) |
| Not applicable or none | 59 (44.7%) |
| Perceived Stress Questionnaire-Index | 0.7 (0.1) [0.4-1.0] |

*Note.* ^a^: including respondent; ^b^: 17 years old and below; ^c^: for 30 minutes or longer in a week, including physical activity at work
